# Tracking Multiple Targets from Multistatic Doppler Radar with Unknown Probability of Detection

**DOI:** 10.3390/s19071672

**Published:** 2019-04-08

**Authors:** Cong-Thanh Do, Hoa Van Nguyen

**Affiliations:** 1 School of Electrical Engineering, Computing, and Mathematical Sciences, Curtin University, Bentley, WA 6102, Australia; 2 School of Computer Science, The University of Adelaide, Adelaide, SA 5005, Australia; hoavan.nguyen@adelaide.edu.au

**Keywords:** multitarget tracking, multistatic Doppler radar, unknown detection probability, Bayes recursion, labeled RFS, GLMB, bootstrapped detection probability

## Abstract

The measurements from multistatic radar systems are typically subjected to complicated data association, noise corruption, missed detection, and false alarms. Moreover, most of the current multistatic Doppler radar-based approaches in multitarget tracking are based on the assumption of known detection probability. This assumption can lead to biased or even complete corruption of estimation results. This paper proposes a method for tracking multiple targets from multistatic Doppler radar with unknown detection probability. A closed form labeled multitarget Bayes filter was used to track unknown and time-varying targets with unknown probability of detection in the presence of clutter, misdetection, and association uncertainty. The efficiency of the proposed algorithm was illustrated via numerical simulation examples.

## 1. Introduction

Initiated from the 1930s from a very simple device for aircraft detection, the radar has been developed into complicated systems in both civilian applications and modern warfare for the purposes of prevention as well as interception strategies [1]. As implied from its name, RADAR (RAdio Detection And Ranging), the main purposes of this system are not only detecting targets or obstacles but also estimating several parameters like velocity, range, and bearing of these targets from electromagnetic signals [2]. Since target tracking for radar is subjected to clutter (caused by environment) and various distortions (due to the signal propagation), the estimation accuracy and detection probability are limited. An improvement on tracking performance for radar is the use of a multistatic radar system (MRS). This system is equipped with multiple transmitting and receiving antenna pairs, which are spatially distributed in a large region of surveillance comparing to the antenna sizes [3] to maximize the estimate accuracy and probability of detection.

The large geographical separation of transmitters and receivers is an essential feature of the MRS [4] and leads to a notable increase in potentially useful information. Since different transmitter-receiver pairs can detect targets at different aspects, each target is observed from multi-directional perspectives and its accurate recognition is significantly enhanced [1]. Other benefits of using MRS are the increase of resolution capability, as well as jamming and clutter resistances [5]. In addition, the spatial distribution of radars prevents the whole radar system from physically being destroyed from attacks.

The more antennas in the radar system, the more complex the target tracking will be. First, the whole system needs lines-of-sight between radar stations and targets for information fusion, not to mention requirements on synchronization, phasing and the positioning of stations, and transmission of reference frequencies and signals [5]. Second, in multitarget tracking, there are generic challenges, such as unknown and randomly time-varying number of the targets, detection uncertainty, clutter, and data association uncertainty [6], as well as nonlinearity and low observability of the Doppler measurement [7,8]. Both locations and velocities of moving targets must be monitored from track history to heading continuously for consistent detection. Obviously, the detection using Doppler effect from stationary radars can only be matched to moving targets, since the reflected signals from motionless targets (or very slow moving ones) are practically identical to the frequencies of the transmitted signals. The multitarget tracking problem using a multistatic radar system is, therefore, a paramount challenge but a greatly attractive field of information fusion study.

The core of multitarget tracking algorithms is to filter the target state from measurements such that the produced estimates of the multitarget state are as close to the ground truth as possible [9]. Currently, three notable approaches to the problem of multitarget tracking have been proposed, namely: the Joint Probabilistic Data Association Filter (JPDAF); Multiple Hypothesis Tracking (MHT); and Random Finite Set (RFS) [6]. Different from the first two, which attempt to modify single target tracking algorithms to deal with multiple targets via data association, the RFS approach uses several fundamental concepts in estimation theory, such as multitarget estimation error and Bayes optimality to provide a top-down formulation of multitarget estimation [10,11].

The RFS approach attracts the attention of the data fusion research community in recent years thank to its advantages in this field of study [9]. Since the inception of RFS, a suite of multitarget filters have been developed, e.g., the Probability Hypothesis Density (PHD) filter [12,13], Cardinalized PHD (CPHD) filter [14,15], multi-Bernoulli filters [16,17], Generalized Labeled Multi-Bernoulli (GLMB) filter [18,19], Labeled Multi-Bernoulli filter [20], and multi-scan GLMB filter [21]. The most advanced RFS-based algorithm, the GLMB filter, can output target tracks and can be implemented with linear complexity in the number of measurements using Gibbs sampling [22]. More recently, the RFS method has been applied to tracking from merged measurements [23], extended targets [24], maneuvering targets [25], track-before-detect [26,27], computer vision [28,29], sensor scheduling [30,31], distributed fusion [32], field robotics [33], cell biology [34], and machine learning [35]. Particularly, as demonstrated via the tracking of more than one million targets per scan in clutter and in real-time [36], this filter is currently considered the most effective multitarget tracker [9].

Although many RFS-based filters such as the PHD and multi-Bernoulli filters, have been applied to Doppler measurements [37,38], they do not produce target tracks and are crude approximations to the Bayes multitarget filter. In addition, most of these filters consider the probability of detection as a known parameter. However, this consideration is impractical because in practice this is rarely the case and the probability of detection need to be manually tuned on a trial-and-error basis. Based on the RFS approach, this paper proposes a solution to the challenges in using Doppler-only measurement for multiple marine vessels tracking with unknown detection parameter a priori in a timely manner. This is an extension of our previous work, reported in Reference [39]. Particularly, in Reference [39], the probability of detection was assumed to be known in advance, while in this work, we did not assume that this parameter was known a priori. When this parameter is unknown, assuming a known probability of detection will degrade the filtering performance. The tracking problem for unknown detection probability, in the present work, is much more difficult and complicated than that of the previous one. The proposed filter in current work solved not only the problem of multiple targets tracking, as mentioned in the previous version, but also the problem of the unknown detection probability by estimating it on the fly.

Apart from the introduction, the paper includes the following four sections. First, the background of target detection using multistatic Doppler measurement, along with the labeled RFS approach to multitarget tracking, is presented. Second, the GLMB filter applied to the multitarget tracking multistatic Doppler measurement with bootstrapped unknown detection profile is shown. Third, validation of the proposed solution is illustrated by numerical simulations on marine ships, followed by some concluding remarks.

## 2. Multistatic Doppler Measurement Model

**Figure 1:**
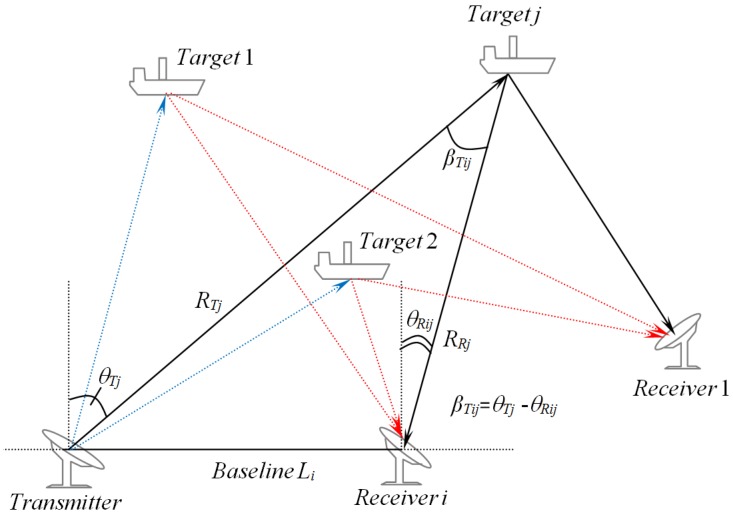
Multitarget tracking using a multistatic radar system (MRS) scenario.

Typically, a passive multistatic radar system is realized with either one receiver and multiple transmitters or a single transmitter combined with multiple radar receivers. Although the latter is more costly than the former, its has better observability [40]. An illustration of a multitarget tracking using a multistatic radar scenario with one transmitter and several receivers is shown in Figure 1. The time difference of arrival (TDOA) between the transmitter-*i*th receiver and transmitter-*j*th target-*i*th receiver is proportional to the different range: $R_{Tj}+R_{Rij}-L_{i}$, where $R_{Tj},R_{Rij},\mathrm{and}\;L_{i}$ are the distances of the transmitter-*j*th target, *j*th target - *i*th receiver, and transmitter- *i*th receiver, respectively. Several solutions for the problems of localization and target tracking via radar signals have been proposed in the literature by means of measuring TDOA or the angle of arrival $\theta_{Rij}$, or the Doppler shift of the received echo [4]. For instance, some methods for single target localization using target range and bearing, range and Doppler measurements and Doppler-only measurements are proposed in Reference [41,42,43], and [44], respectively. In addition, the combination of range and range rate measurements—as well as the combination of range, range rate, and elevation measurements—for multisensor multitarget tracking has been presented in Reference [40].

All the conventional methods using multistatic radar-based target tracking consider the probability of target detection to be known a priori, while it is typically unknown and time-varying [11]. Such an assumption leads to biased estimation or even complete corruption of filtering results since this parameter directly influences observability of the target. Recent proposed methods for tackling unknown detection probability, (see, [45,46,47] for instance), do not produce target tracks. Tracking multiple targets using a multistatic radar system is still an attractive field of study.

Taking advantage of the MRS by using a type of MRS, like the multistatic Doppler radar system, has been used for tracking multiple targets with high accuracy [5,48]. In this work, we considered a multistatic passive Doppler radar system consisting of one cooperative transmitter and two spatially stationary distributed receivers (see Figure 1). The Doppler-only measurement method to track targets based on the RFS approach will also be proposed for further use of the tracking algorithm.

By using the Doppler effect, the velocity of the target is calculated from analyzing the pulses of radio signals which are emitted from the transmitter, bouncing off the target then reflecting the receiver [49]. The Doppler measurement of a target with the state $x_{k}$ at the *s*th receiver is given by:

(1) $$\begin{array}{cc} z_{k}^{\left(s\right)} & =-\nu_{k}^{T}\left(\frac{p_{k}-p_{r}^{\left(s\right)}}{\left|\right|p_{k}-p_{r}^{\left(s\right)}\left|\right|}+\frac{p_{k}-p_{t}}{\left|\right|p_{k}-p_{t}\left|\right|}\right)\frac{f_{t}}{c}+w_{k},\\ & ≜h_{k}\left(x_{k}\right)+w_{k} \end{array}$$

in which the target position $p_{k}={\left[\mu_{k},\lambda_{k}\right]}^{T}$ and velocity $\nu_{k}={\left[\dot{\mu_{k}},{\dot{\lambda }}_{k}\right]}^{T}$ are measured in longitudinal and latitudinal coordinates. $p_{t}={\left[\mu_{t},\lambda_{t}\right]}^{T}$ is the transmitter location, $p_{r}^{\left(s\right)}={\left[\mu_{r}^{\left(s\right)},\lambda_{r}^{\left(s\right)}\right]}^{T}$ is the *s*th-receiver position; *T* denotes the transpose operation; $w_{k}$ is zero-mean Gaussian noise with covariance *Q*, $w_{k}∼N\left(0;Q_{k}\right)$; and $f_{t}$ and *c* are the emitted signal frequency of the transmitter and the speed of light, respectively.

Since targets can move in different directions with nonlinear dynamics, and the information collected from Doppler radar is subjected to environmental noise, the measurement in Equation (1) is highly nonlinear.

Several methods have been proposed to tackle those aforementioned challenges using target range and azimuthal direction [41,42], Range-Doppler maps [43], and Doppler-only measurements [44]. However, they are either applied to detect a single target or estimate target positions without producing target tracks in which the probability of target detection is assumed to be known a priori. Hence, a proper method that can produce target tracks with unknown detection probability needs to be considered.

## 3. Background

### 3.1. Labeled RFS

The key point in target tracking is using information collected from sensors to jointly estimate the number of targets and their states, as well as the target trajectories. However, both the number of the targets and their states in a multitarget system randomly vary with time, thus it is difficult to follow the target trajectories. Obviously, using vectors to represent multitarget state is insufficient, since the targets in a multitarget state are unordered and can be changed over time. A discussion about vector and finite set representations of multitarget state, given in Reference [16], has shown that a finite set representation is the most appropriate from an estimation viewpoint. The most appropriate model for multitarget state is, therefore, an RFS [10].

Individual targets in a multitarget state can distinguished by their labels [10]. Indeed, the key concept here is the assignment of a uniquely identifying track label *ℓ* to each kinematic target state *x* (i.e., $x=\left(x,\ell \right)$). Moreover, this assignment, $x=\left(x,\ell \right)$, can occur only once in the finite subset *X* [9], and the labeled finite subset is now denoted as *X*. By using this concept, the so-called labeled RFS proposed by Vo and Vo [18,19], the problem of the indistinguished targets can be solved [11].

**Definition** **1.** *[18] Let L:X×L→L be the projection L(x;ℓ)=ℓ, and hence $L\left(X\right)=\left\{L\left(x\right):x\in X\right\}$ is the set of labels of X. A labeled RFS with space X and (discrete) label space L is an RFS on X×L such that each realization X has distinct labels, i.e., |L(X)|=|X|.*

It should be mentioned that the unlabeled version of a labeled RFS is obtained by simply discarding the labels. Unlabeled multitarget filtering formulation does not have the mechanism to address target tracks and, as a result, heuristic techniques are needed to produce target tracks [9].

In this paper, the unlabeled states are denoted by normal-faced letters to distinguish from labeled ones, which are denoted by the bold-faced letters (i.e., x,y,X,Y and **x,y,X,Y**), in which the single target state and multitarget state are denoted by lower-case letters and upper-case letters, respectively. The spaces corresponding to variables are symbolized by blackboard bold letters (e.g., X,Z,L, etc.). The inclusion function $1_{S}\left(\cdot \right)$ and the Kronecker delta function $\delta_{S}\left(\cdot \right)$ for a set *S* are given to support arbitrary arguments *X* (e.g., sets, vectors, and integers) as follows [19]:

$$1_{S}\left(X\right)=\left\{\begin{array}{cc} 1 & \mathrm{if}\;X\subseteq S\\ 0 & \mathrm{otherwise}, \end{array}\right.\;\delta_{S}\left(X\right)=\left\{\begin{array}{cc} 1 & \mathrm{if}\;X=S\\ 0 & \mathrm{otherwise}. \end{array}\right.$$

The class of finite subsets of *S* is represented by $F\left(S\right)$, and the inner product is denoted by:

$$\langle f,g\rangle ≜f\left(x\right)g\left(x\right)dx.$$

The sequence of variables $X_{i},X_{i+1},\ldots ,X_{j}$ is abbreviated as $X_{i:j}$, and the cardinality of a finite set *X* is denoted by |X|.

### 3.2. Bayesian Multitarget Recursion

In classical Bayesian recursion, two assumptions are given, as follows [50]: (i) The hidden states follow a first-order Markov process on the state space according to a transition density $f_{k|k-1}\left(x_{k}|x_{k-1}\right)$, and (ii) the observations are conditionally independent of the given states and are characterized by a likelihood $g_{k}\left(z_{k}|x_{k}\right)$. By incorporating prior knowledge and observational evidence of a target, the Bayesian recursion has been formulated for the problem of single-target single-measurement systems.

Suppose that, at time *k*, there are $n_{k}$ targets $\left(i.e.,\;\mathrm{the}\;\mathrm{individual}\;\mathrm{states}\;\mathrm{are}\;x_{k,1},\ldots ,x_{k,n_{k}}\right)$, and the number of measurements is $m_{k}$ $(i.e.,\;\mathrm{the}\;\mathrm{individual}\;\mathrm{measurements}\;\mathrm{are}\;z_{k,1},\ldots ,z_{k,m_{k}}$), then the multitarget state and multitarget observation are [19]:

$$\begin{array}{cc} X_{k} & =\left\{x_{k,1},\ldots ,x_{k,n_{k}}\right\}\in F\left(X\times L_{k}\right),\\ Z_{k} & =\left\{z_{k,1},\ldots ,z_{k,m_{k}}\right\}\subset Z. \end{array}$$

By using Bayes theorem, it could be seen that, conditioned on the measurement history $Z_{0:k}=\left(Z_{0},\ldots ,Z_{k}\right)$, all the information on the set of target trajectories $X_{0:k}=\left(X_{0},\ldots ,X_{k}\right)$ can be captured by the multitarget posterior density, which is given recursively for k≥1 as follows:

$$\begin{array}{cc} \pi_{0:k}\left(X_{0:k}|Z_{0:k}\right) & \propto g_{k}\left(Z_{k}|X_{k}\right)f_{k|k-1}\left(X_{k}|X_{k-1}\right)\times \pi_{0:k-1}\left(X_{0:k-1}|Z_{0:k-1}\right), \end{array}$$

in which $g_{k}$ and $f_{k|k-1}$ are the multitarget likelihood function and multitarget transition density to time *k*, respectively. By using $g_{k}$ and $f_{k|k-1}$, the underlying models for detection and false alarms and those for target motions, births, and deaths are encapsulated [19].

Assuming that, at the previous time step k−1, the multitarget state is distributed according to a multitarget density $\pi_{k-1}\left(\cdot |Z_{1:k-1}\right)$. At time k, the measurement $Z_{k}$ is the superposition of detected targets and false measurements and is modeled by $g_{k}\left(\cdot |\cdot \right)$. The multitarget prediction and the multitarget posterior to time *k* is given by the Chapman–Kolmogorov equation and Bayes rule, respectively:

(2) $$\begin{array}{cc} \pi_{k|k-1}\left(X_{k}|Z_{1:k-1}\right) & =\int f_{k|k-1}\left(X_{k}|X_{k-1}\right){\times \pi }_{k-1}\left(X_{k-1}|Z_{1:k-1}\right)\delta X_{k-1}, \end{array}$$

(3) $$\begin{array}{cc} \pi_{k}\left(X_{k}|Z_{1:k}\right) & =\frac{g_{k}\left(Z_{k}|X_{k}\right)\pi_{k|k-1}\left(X_{k}|Z_{1:k-1}\right)}{\int g_{k}\left(Z_{k}|X\right)\pi_{k|k-1}\left(X|Z_{1:k-1}\right)\delta X}. \end{array}$$

Noting that the integral above is a set integral:

$$\int f\left(X\right)\delta X=\sum_{i=0}^{\infty }\frac{1}{i!}\int f\left(\left\{x_{1},\ldots ,x_{i}\right\}\right)d\left(x_{1},\ldots ,x_{i}\right),$$

defined for any function $f:F\left(X\times L\right)\to R.$

### 3.3. Multitarget State Model

**Figure 2:**
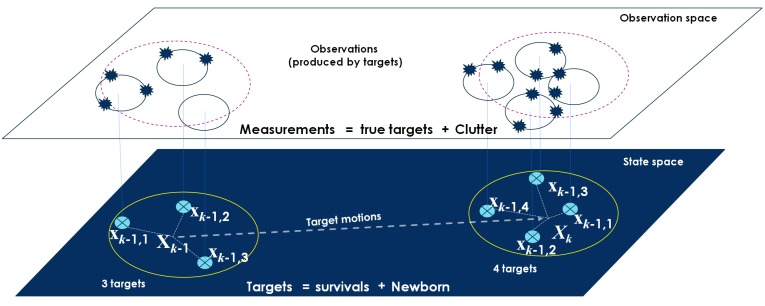
Illustration of the state space model with multitarget state [51].

For the purpose of RFS-based multitarget tracking, the RFS formulation of the standard multitarget dynamic model which captures all targets, including appearance, disappearance, and evolution ones over the time, will be investigated. Let $X_{k-1}$ represent the set of all individual target states at time k−1. Each target state $x_{k-1}$ can survive and evolve to a new state $x_{k}$ at time *k* with survival probability $P_{S,k}\left(x_{k-1}\right)$ or disappear between the time duration of k−1 and *k* with probability $1-P_{S,k}\left(x_{k-1}\right)$, not mention to some new-appear targets at time *k* (see Figure 2). The complete multitarget state $X_{k}$ at time *k* is the superposition of survivals and births [18]:

(4) $$X_{k}=\underset{x_{k-1}\in X_{k-1}}{⋃}S_{k|k-1}\left(x_{k-1}\right)⋃B_{k},$$

in which $S_{k|k}\left(x_{k-1}\right)$ and $B_{k}$ are the labeled RFS of existing target state from time k−1 to time *k* and that of the new born states at time *k*, respectively. The labeled Bernoulli RFS $S_{k|k-1}\left(x_{k-1}\right)$ at current time *k* is generated by a given state $x_{k-1}\in X_{k-1}.$ Since a labeled state $x_{k-1,i}=\left(x_{k-1,i},\ell_{i}\right){\in X}_{k-1}$ can exist and evolve to a new state $x_{k,i}=\left(x_{k,i},\ell_{i}\right)\in X_{k}$ with survival probability $P_{S}\left(x_{k-1,i}\right)$ and probability density $f\left(x_{k,i}|x_{k-1,i},\ell_{i}\right)\delta_{\ell_{i}}\left(\ell_{i}\right)$ (where $f\left(x_{k,i}|x_{k-1,i},\ell_{i}\right)$ is the single target transition kernel); or can disappear with probability $1-P_{S}\left(x_{k-1,i}\right)$, we have [18],

(5) $$f_{S}\left(S|X\right)=\Delta \left(S\right)\Delta \left(X\right)1_{L\left(X\right)}\left(L\left(S\right)\right){\left[\Phi \left(S;\cdot \right)\right]}^{X},$$

where $f_{S}\left(S|X\right)$ is the distribution function of the surviving target set *S* the next time, and:

$$\Phi \left(S;x,\ell \right)=\left\{\begin{array}{cc} P_{S}\left(x_{k-1},\ell \right)f\left(x_{k}|x_{k-1},\ell \right) & \mathrm{if}\;\left(x_{k},\ell \right)\in S\\ 1-P_{S}\left(x_{k-1},\ell \right) & \mathrm{if}\;\ell \notin L\left(S\right) \end{array}\right..$$

The set $B_{k}$ of new-born states is distributed according to [19]:

(6) $$f_{B}\left(Y\right)=\Delta \left(Y\right)\omega_{B}\left(L\left(Y\right)\right){\left[p_{B}\right]}^{Y},$$

where $\omega_{B}$ and $p_{B}$ are given parameters of the multitarget birth density $f_{B,}$ defined on space X×B. Whenever the set *Y* contains an element *y* with $L\left(y\right)\notin B,$ $f_{B}=0.$ The birth model (Equation (6)) includes both labeled Poisson and labeled multi-Bernoulli [18].

The multitarget transition density is given by [19]:

(7) $$f\left(X_{k}|X_{k-1}\right)=f_{S}\left(X_{k}\cap \left(X\times L\right)|X_{k-1}\right)f_{B}\left(X_{k}-\left(X\times L\right)\right).$$

### 3.4. Multitarget Observation Model

Given a target state $x_{k}=\left(x,\ell \right)$, the *s*th receiver can detect this state with probability $p_{D}^{\left(s\right)}\left(x,\ell \right)$ and generates a measurement $z_{k}^{\left(s\right)}\in Z_{k}^{\left(s\right)}$ with likelihood $g^{\left(s\right)}\left(z_{k}^{\left(s\right)}|x,\ell \right)$, or being missed with probability $1-p_{D}^{\left(s\right)}\left(x,\ell \right)$. Considering the multiple target scenario, the multitarget observation $Z_{k}^{\left(s\right)}=\left\{z_{k,1}^{\left(s\right)},\ldots ,z_{k,m_{k}}^{\left(s\right)}\right\}$ at time *k* is the superposition of the detected targets and Poisson clutter with intensity function κ [10], meaning that the information collected by radar includes false alarms. The probability of target detection, therefore, has a significant effect on the performance of the tracker. This conclusion is indeed true to RFS-based recursive Bayesian multitarget filtering.

Assuming that condition on *X*, detections are independent of each other and clutter, the multitarget likelihood function of sensor *s* is given as follows [18,19]:

(8) $$g^{\left(s\right)}\left(Z^{\left(s\right)}|X\right)\propto \sum_{\theta^{\left(s\right)}\in \Theta^{\left(s\right)}}1_{\Theta^{\left(s\right)}\left(L\left(X\right)\right)}\left(\theta^{\left(s\right)}\right)\times \prod_{\left(x,\ell \right)\in X}\psi_{Z^{\left(s\right)}}^{\left(s,\theta^{\left(s\right)}\left(\ell \right)\right)}\left(x,\ell \right),$$

where $\Theta^{\left(s\right)}$ is the set of the positive 1-1 maps $\theta^{\left(s\right)}:L\to \left\{0:|Z^{\left(s\right)}|\right\}$, i.e., maps such that no distinct arguments are mapped to the same positive value, $\Theta^{\left(s\right)}\left(I\right)$ is the subset of $\Theta^{\left(s\right)}$ with domain *I*; and:

(9) $$\psi_{z_{1:M^{\left(s\right)}}}^{\left(s,j\right)}\left(x,\ell \right)=\left\{\begin{array}{cc} \frac{p_{D}^{\left(s\right)}\left(x,\ell \right)g^{\left(s\right)}\left(z_{j}|x,\ell \right)}{\kappa^{\left(s\right)}\left(z_{j}\right)}, & \mathrm{if}\;j=1:M^{\left(s\right)}\\ 1-p_{D}^{\left(s\right)}\left(x,\ell \right) & \mathrm{if}\;j=0 \end{array}\right.,$$

where $M^{\left(s\right)}$ is the number of measurements from sensor *s*.

The map $\theta^{\left(s\right)}$ specifies which object generated which detection from sensor *s*, i.e., target *ℓ* generates detection $z_{\theta \left(\ell \right)}\in Z^{\left(s\right)}$ with undetected targets assigned to 0. $\theta^{\left(s\right)}$ is 1–1 on $\left\{\ell :\theta^{\left(s\right)}\left(\ell \right)>0\right\}.$ The positive 1-1 property means that each distinct label is only mapped to a distinct positive value. Therefore, this property ensures that any detection in $Z^{\left(s\right)}$ is assigned to, at most, one target.

Followed by Reference [52], the multisensor likelihood is given by:

(10) $$g\left(Z|X\right)=\prod_{s=1}^{M}g^{\left(s\right)}\left(Z^{\left(s\right)}|X\right)\propto \sum_{\theta \in \Theta }1_{\Theta \left(L\left(X\right)\right)}\left(\theta \right)\times \prod_{\left(x,\ell \right)\in X}\psi_{Z}^{\left(\theta \left(\ell \right)\right)}\left(x,\ell \right),$$

where:

$$\begin{array}{cc} Z & =Z^{\left(1:N\right)},\\ \theta & =\theta^{\left(1:N\right)},\\ \Theta & =\Theta^{\left(1\right)}\times \ldots \times \Theta^{\left(N\right)}\left(I\right),\\ 1_{\Theta \left(I\right)}\left(\theta \right) & =\prod_{s=1}^{N}1_{\Theta^{\left(s\right)}\left(I\right)}{\left(\theta \right)}^{\left(s\right)},\\ \psi_{Z}^{\left(j^{\left(1:N\right)}\right)}\left(x,\ell \right) & =\prod_{s=1}^{N}\psi_{Z^{\left(s\right)}}^{\left(s,j^{\left(s\right)}\right)}\left(x,\ell \right). \end{array}$$

*N* is the sensor observation set. It could be seen that the form of the multisensor likelihood function and that of the single sensor likelihood function are identical.

Basically, a GLMB density can be rewritten in the following form [18,19]:

(11) $$\pi \left(X\right)=\Delta \left(X\right)\sum_{\xi \in \Xi }\sum_{I\subseteq L}\omega^{\left(I,\xi \right)}\delta_{I}\left[L\left(X\right)\right]{\left[p^{\left(\xi \right)}\right]}^{X},$$

where each $\xi =\left(\theta_{1:k}\right)\in \Xi$ represents each history of multisensor association maps, each $\omega^{\left(I,\xi \right)}$ is non-negative satisfying $\sum_{\xi \in \Xi }\sum_{I\subseteq L}\omega^{\left(I,\xi \right)}=1$, and each $p^{\left(\xi \right)}\left(\ell \right)$ is a probability density on X. The cardinality distribution, the existence probability, and probability density of track ℓ∈L are, respectively,

(12) $$\begin{array}{cc} Pr\left(|X|=n\right) & =\sum_{\xi \in \Xi }\sum_{I\subseteq L}\delta_{n}\left[|I|\right]\omega^{\left(I,\xi \right)}, \end{array}$$

(13) $$\begin{array}{cc} r\left(\ell \right)= & \sum_{\xi \in \Xi }\sum_{I\subseteq L}1_{I}\left(\ell \right)\omega^{\left(I,\xi \right)}, \end{array}$$

(14) $$\begin{array}{cc} p\left(x,\ell \right)= & \frac{1}{r\left(\ell \right)}\sum_{\xi \in \Xi }\sum_{I\subseteq L}1_{I}\left(\ell \right)\omega^{\left(I,\xi \right)}p^{\left(\xi \right)}\left(x,\ell \right). \end{array}$$

Given the GLMB density (Equation (11)), an intuitive multi-object estimator is the multi-Bernoulli estimator [52]. Given a prescribed threshold existence probability, this estimator determines the set of labels L⊆L which have higher existence probabilities the threshold, then the states of the objects will be estimated by using mode/mean estimates from the densities p(·,ℓ),ℓ∈L.

### 3.5. Multisensor GLMB Recursion

Under the standard multitarget dynamic and observation models, the GLMB filter is an analytic solution to the Bayes single-sensor multitarget filter [18]. In addition, the form of the multisensor likelihood function is identical to that of a single-sensor likelihood function, as previously concluded, thus a suggestion on a multisensor GLMB is given as follows [52]: Given the filtering density (Equation (11)) at time *k*, the filtering density at time k+1 is given by:

(15) $$\pi_{+}\left(X_{+}\right)\propto \Delta \left(X_{+}\right)\sum_{I\xi ,I_{+},\theta_{+}}\omega^{\left(I,\xi \right)}\omega_{Z_{+}}^{\left(I,\xi ,I_{+},\theta_{+}\right)}\delta_{I_{+}}\left[L\left(X_{+}\right)\right]{\left[p_{Z_{+}}^{\left(\xi ,\theta_{+}\right)}\right]}^{X_{+}},$$

where $I\in F\left(L\right),\xi \in \Xi ,I_{+}\in F\left(L_{+}\right),\theta_{+}\in \Theta_{+}\left(I_{+}\right)$, and:

(16) $$\begin{array}{cc} \omega_{Z_{+}}^{\left(I,\xi ,I_{+},\theta_{+}\right)}= & 1_{\Theta_{+}\left(I_{+}\right)}\left(\theta_{+}\right){\left[1-{\bar{P}}_{S}^{\left(\xi \right)}\right]}^{I-I_{+}}{\left[{\bar{P}}_{S}^{\left(\xi \right)}\right]}^{I\cap I_{+}}{\left[1-r_{B,+}\right]}^{B_{+}-I_{+}}r_{B,+}^{B_{+}\cap I_{+}}{\left[{\bar{\psi }}_{Z_{+}}^{\left(\xi ,\theta_{+}\right)}\right]}^{I_{+}} \end{array}$$

(17) $$\begin{array}{cc} {\bar{P}}_{S}^{\left(\xi \right)}\left(\ell \right)= & \langle p^{\left(\xi \right)}\left(\cdot ,\ell \right),P_{S}\left(\cdot ,\ell \right)\rangle \end{array}$$

(18) $$\begin{array}{cc} {\bar{\psi }}_{Z_{+}}^{\left(\xi ,\theta_{+}\right)}\left(\ell_{+}\right)= & \langle {\bar{p}}_{+}^{\left(\xi \right)}\left(\cdot ,\ell_{+}\right),\psi_{Z_{+}}^{\left(\theta_{+}\left(\ell_{+}\right)\right)}\left(\cdot ,\ell_{+}\right)\rangle \end{array}$$

(19) $$\begin{array}{cc} {\bar{p}}_{+}^{\left(\xi \right)}\left(x_{+},\ell_{+}\right)= & 1_{L}\left(\ell_{+}\right)\frac{\langle P_{S}\left(\cdot ,\ell_{+}\right)f_{+}\left(x_{+}|\cdot ,\ell_{+}\right),p^{\left(\xi \right)}\left(\cdot ,\ell_{+}\right)\rangle }{{\bar{P}}_{S}^{\left(\xi \right)}\left(\ell_{+}\right)}+1_{B_{+}}\left(\ell_{+}\right)p_{B,+}\left(x_{+},\ell_{+}\right) \end{array}$$

(20) $$\begin{array}{cc} p_{Z_{+}}^{\left(\xi ,\theta_{+}\right)}\left(x_{+},\ell_{+}\right)= & \frac{{\bar{p}}_{+}^{\left(\xi \right)}\left(x_{+},\ell_{+}\right)\psi_{Z_{+}}^{\left(\theta_{+}\left(\ell_{+}\right)\right)}\left(x_{+},\ell_{+}\right)}{{\bar{\psi }}_{Z_{+}}^{\left(\xi ,\theta_{+}\right)}\left(\ell_{+}\right)} \end{array}$$

Obviously, by rewriting Equation (15) as a sum over $I_{+},\xi ,\theta_{+}$ with weights:

(21) $$\omega_{+}^{\left(I_{+},\xi ,\theta_{+}\right)}\propto \sum_{I}\omega^{\left(I,\xi \right)}\omega_{Z_{+}}^{\left(I,\xi ,I_{+},\theta_{+}\right)},$$

Equation (15) will have the same form with Equation (11). It means that the GLMB recursion for a single sensor can be applied to the problem of multiple sensors, and only the components $\left(I_{+},\xi ,\theta_{+}\right)$ with weights $\omega_{+}^{\left(I_{+},\xi ,\theta_{+}\right)}$ need to be forwardly propagated at the next iteration.

### 3.6. Adaptive to Unknown Detection Probability

Normally, the probability of detection is assumed to be known a priori, however, this assumption is impractical. Therefore, the parameter $p_{D}^{\left(s\right)}$ in Equation (9) much be estimated simultaneously with the update steps. It has been illustrated in Reference [28] that, with a specially chosen state space, the GLMB filter can be applied to solve the problem of tracking targets under the unknown probability of detection [47].

**Figure 3:**
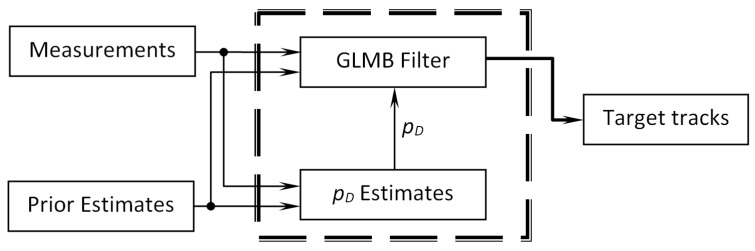
$p_{D}$-bootstrapped Generalized Labeled Multi-Bernoulli (BpD-GLMB) filter diagram.

In this work, a new method called BpD-GLMB for tracking multitargets was proposed to estimate the unknown probability of target detection and produce target tracks online. Specifically, the unknown probability of detection parameter $p_{D}$ was estimated separately, then bootstrapped into the state-of-the-art GLMB filter for target tracking (see Figure 3).

Inspired by Reference [53,54], this paper proposes using the pD-cardinalized PHD filter [11] to separately estimate the probability of detection, and then bootstrapped this parameter into the update stage of GLMB filter.

The idea of accommodating the unknown and non-homogeneous detection probability by incorporating this parameter into the target state variable has been proposed in Reference [11], in which each usual kinematic state *x* is replaced by an augmented state $\overset{˚}{x}=\left(a,x\right)$, where 0≤a≤1 is the unknown target detection probability of *x*. Obviously, the augmented state encompasses both the original kinematic target state *x* and the corresponding unknown and state-dependent detection probability (represented by *a*). Consequently, the augmented multitarget state has the form:

(22) $$\overset{˚}{X}=\left\{{\overset{˚}{x}}_{1},\ldots ,{\overset{˚}{x}}_{n}\right\}\;=\left\{\left(a_{1},x_{1}\right),\ldots ,\left(a_{n},x_{n}\right)\right\}\;,$$

where *n* is the number of the target state, and the corresponding set integral has the form [11]:

(23) $$\int \overset{˚}{f}\left(\overset{˚}{X}\right)\delta \overset{˚}{X}=\sum_{n\geq 0}\frac{1}{n!}\overset{˚}{f}\left(\left\{{\overset{˚}{x}}_{1},\ldots ,{\overset{˚}{x}}_{n}\right\}\right)d{\overset{˚}{x}}_{1}\ldots d{\overset{˚}{x}}_{n}.$$

Since the probability of detection is unknown a priori, the filter should estimate this parameter such that the measurements are well adapted with the underlying target/object model [47]. By using the augmented state, the augmented space is given by:

$$\overset{˚}{X}=\bar{X}\times X,$$

where $\bar{X}=\left[0,1\right]$ and $X=R^{n_{x}}$ denote the spaces of detection probability and target kinematics, respectively. The augmented detection probability and single target likelihood function are described as follows:

(24) $$\begin{array}{cc} {\overset{˚}{p}}_{D}^{\left(s\right)}\left(\overset{˚}{x}\right) & ={\overset{˚}{p}}_{D}^{\left(s\right)}\left(a,x\right)≜a \end{array}$$

(25) $$\begin{array}{cc} {\overset{˚}{g}}_{z}^{\left(s\right)}\left(\overset{˚}{x}\right) & ={\overset{˚}{g}}_{z}^{\left(s\right)}\left(a,x\right)≜g_{z}^{\left(s\right)}\left(x\right). \end{array}$$

Here, it is assumed that regardless of undetectability, a target will generate the same measurement. For the purposes of using RFS filters to tackle unknown probability of detection, the simple substitution of variables has been proposed in Reference [11] as follows: While keeping the likelihood function unchanged, the target state *x* and detection probability $p_{D}^{\left(s\right)}\left(x\right)$ are replaced by $\left(a,x\right)$ and *a*, respectively. Consequently, the integral ∫·dx is substituted by ${\;\int \int \;}_{0}^{1}\cdot dadx$.

### 3.7. Implementation

Because the number of terms in a GLMB updated multitarget density grows exponentially with time, practically only the terms with largest weights should be retained to limit the exhaustive evaluation of all terms. This work minimizes the $L_{1}$ approximation error [19] and can be formulated as a ranked implementation multi-dimensional assignment problem.

Although the K-short path algorithm can be used to compute the best terms of the prediction, it is easy for tracking loss unless the number of predicted terms is large enough. An alternative algorithm to mitigate this problem is the use of unscented transformation [55]. By using this algorithm, the predictions for target births and survivals are performed separately and then combined afterward [19]. For the purpose of reducing the cost of computation in the update state, measurement gating and pruning are applied in the GLMB filter. For the single-sensor case, two techniques, called the Murty’s ranked assignment algorithm and Gibbs sampling, have been proposed to perform the truncation without having to propagate all the components [19,22]. While the Murty’s algorithm can be used to determine a given number of highest weighted components of the multi-object filtering density without exhaustively generating all possible mappings, the Gibbs sampler can generate the significant components of the multi-object filtering density for a large number of targets to be tracked. Since the problem of tracking with Doppler measurements in this work requires multiple sensors, both the Murty’s algorithm and Gibbs sampler implementation should be considered. However, the implementation of the two sensor GLMB filter developed in Reference [56] using Murty’s algorithm showed that it has a cubic complexity in the product of the number of measurements from the sensors. Based on the extension of the Gibbs sampler implementation to multiple sensors proposed in Reference [52], Gibbs sampling is chosen as the most appropriate implementation for this problem. As a proof of concept of how GLMB filter addresses multistatic Doppler measurements, the simpler “iterated corrector” implementation that applies single sensor updates once for each sensor, in turn, has been used. This strategy would yield the exact solution if all components of the multitarget filtering density are kept.

## 4. Numerical Studies

In the present work, the problem of tracking 10 nonlinear birth-and-death time-varying marine ships was considered under the missed-detection-and-clutter observations and unknown detection probability. Consider the scenario illustrated in Figure 1, where we adopted the generic constant-turn model to better accommodate the unpredictable maneuvering behaviors of targets. The target state at time *k* is modeled using a 5-D vector $x_{k}={\left[p_{k}^{x},\;{\dot{p}}_{k}^{x},\;p_{k}^{y},\;{\dot{p}}_{k}^{y},\psi_{k}\right]}^{T}$, comprising of its *x*-coordinate, *x*-velocity, *y*-coordinate, *y*-velocity, and course. Hence, the marine ship dynamic model can be expressed as follows:

(26) $$x_{k}=F_{k|k-1}\left(x_{k-1}\right)+Gn_{k},$$

where:

(27) $$F_{k|k-1}\left(x_{k-1}\right)=\left[\begin{array}{ccccc} 1 & \frac{\sin\left(\Delta \psi_{k-1}\right)}{\psi_{k-1}} & 0 & \frac{\cos\left(\Delta \psi_{k-1}\right)-1}{\psi_{k-1}} & 0\\ 0 & \cos\left(\Delta \psi_{k-1}\right) & 0 & -\sin\left(\Delta \psi_{k-1}\right) & 0\\ 0 & -\frac{\cos\left(\Delta \psi_{k-1}\right)-1}{\psi_{k-1}} & 1 & -\frac{\sin\left(\Delta \psi_{k-1}\right)}{\psi_{k-1}} & 0\\ 0 & \sin\left(\Delta \psi_{k-1}\right) & 0 & \cos\left(\Delta \psi_{k-1}\right) & 0\\ 0 & 0 & 0 & 0 & 1 \end{array}\right]x_{k-1};\;G=\left[\begin{array}{ccc} \frac{\Delta^{2}}{2} & 0 & 0\\ \Delta & 0 & 0\\ 0 & \frac{\Delta^{2}}{2} & 0\\ 0 & \Delta & 0\\ 0 & 0 & \Delta \end{array}\right].$$

In which, Δ is the sampling period, and $n_{k}={\left[n_{k}^{\dot{x}},n_{k}^{\dot{y}},n_{k}^{\psi }\right]}^{T}$ is a zero-mean Gaussian noise vector of velocities and course noise components. $\sigma_{\dot{x}}=\sigma_{\dot{y}}$$=\sigma_{v}$ is the standard deviation (std.) of the velocity noise, and $\sigma_{\psi }$ is the std. of the course noise. Note that latitude, longitude, and course are measured in degrees (${}^{\circ }$), while distance, velocity, and time are calculated in nautical miles (*M*), i.e., knots (kn), and hours (h), respectively.

Remark1: The target model with transition matrix $F\left(\psi \right)$ given in Equation (27) is based on the assumption that the surveillance area is located far enough from the North and South Poles.

**Figure 4:**
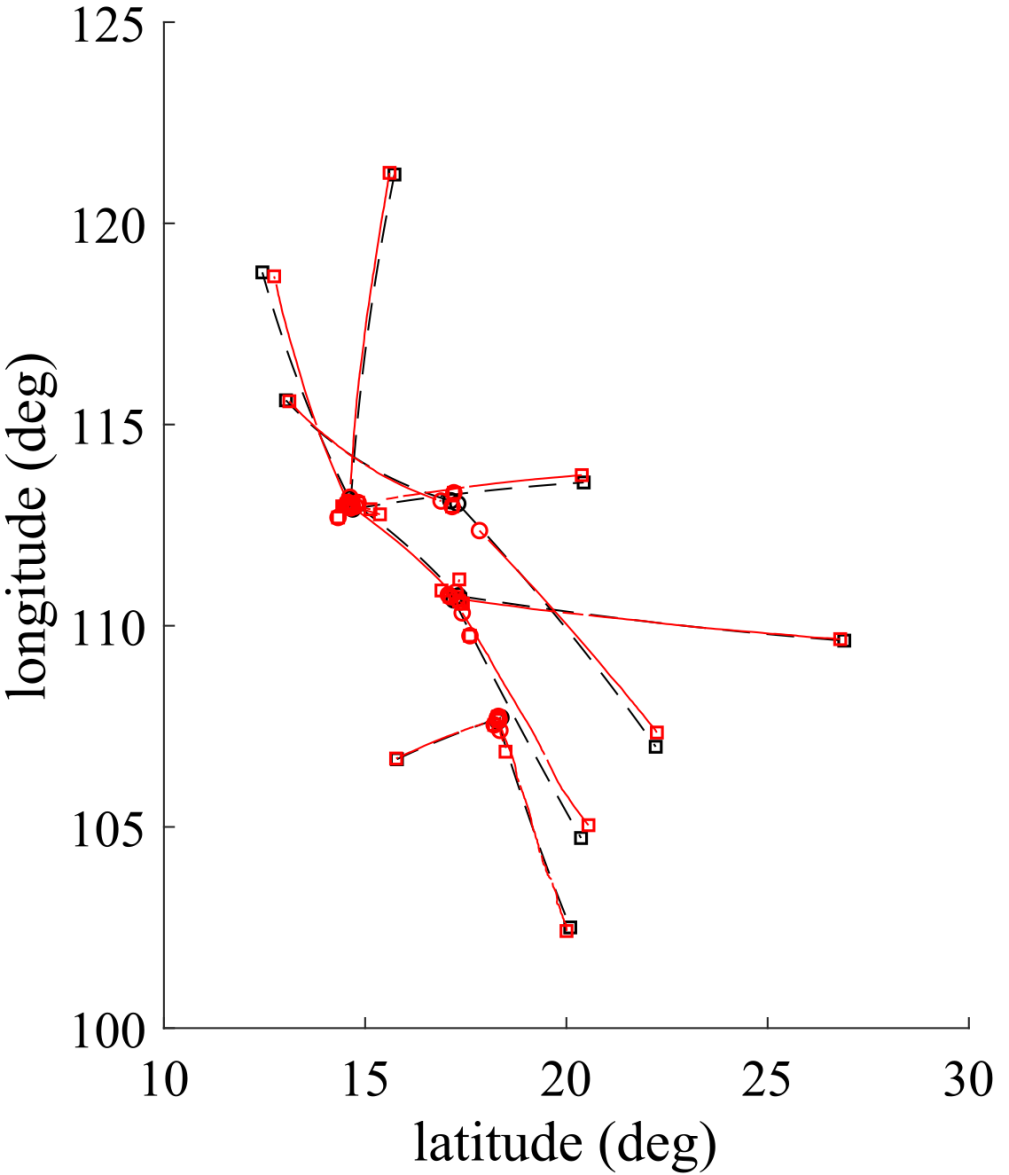
Multitarget ground truths (black) versus its tracked targets (red).

**Figure 5:**
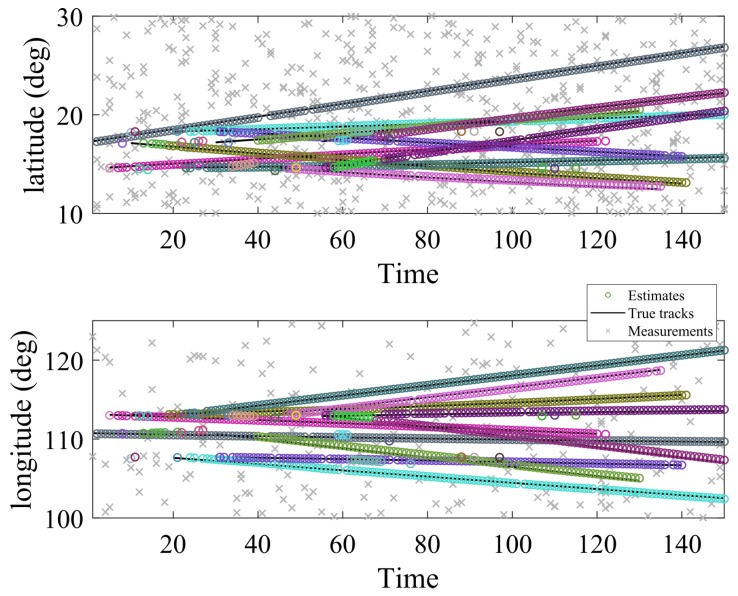
Multitarget tracking in latitude and longitude.

The target model parameters, as well as the birth parameters, are given in Table 1. The multitarget scenario is conducted in the surveillance region $\left[10{}^{\circ }N\;\;30{}^{\circ }N,100{}^{\circ }E\;125{}^{\circ }E\right]$ with a total of 10 targets, which are random in positions, velocities, and the number of appearance (as in Table 2). Target ground truths and tracking results are depicted in Figure 4. By using BpD-GLMB filter, the results of 2-D coordinates target tracks are shown in Figure 5. The birth process is assumed as labeled Multi-Bernoulli RFS with parameters $f_{B}\left(x\right)={\left\{\omega_{B}^{\left(i\right)},p_{B}^{i}\right\}}_{i=1}^{4}$ where the common existence probabilities $\omega_{B}^{\left(1,2\right)}=0.01$ and $\omega_{B}^{\left(3,4\right)}=0.02$ and $p_{B}^{\left(i\right)}\left(x\right)=N\left(x,{\hat{x}}_{B}^{\left(i\right)},P_{B}\right)$ with:

$$\begin{array}{cc} {\hat{x}}_{B}^{\left(1\right)} & ={\left[17.20{}^{\circ }N,0,110,7{}^{\circ }E,0,0\right]}^{T}\\ {\hat{x}}_{B}^{\left(2\right)} & ={\left[14.60{}^{\circ }N,0,113.0{}^{\circ }E,0,0\right]}^{T}\\ {\hat{x}}_{B}^{\left(3\right)} & ={\left[17,20{}^{\circ }N,0,113;0{}^{\circ }E,0,0\right]}^{T}\\ {\hat{x}}_{B}^{\left(4\right)} & ={\left[18.30{}^{\circ }N,0,107,70{}^{\circ }E,0,0\right]}^{T}; \end{array}$$

and:

$$P_{B}=diag{\left(\left[2.0^{\prime }N,30\left(kn\right),2.0^{\prime }E,30\left(kn\right),6\pi /180\left(rad/s\right)\right]\right)}^{2}.$$

Ten targets were assumed to be distributed around the birth model with the closest and farthest latitudinal distances being 2.85 km and 10.73 km, and the corresponding values for longitudinal distances being 2.6 and 10.6 km, respectively. The absolute velocities of the targets were assumed to be varied from 2 to 32 kn (approximately 3.5 to 60 km/h). The assumptions on positions of the multistatic passive Doppler radar transmitter and receivers and transmit frequency $f_{t}$ are given in Table 3.

The measurement space for each receiver is [−200 Hz, 200 Hz], and the measurement noise $w_{k}$ is zero-mean Gaussian noise with the covariance $Q_{k}=diag$([0.5 ${\mathrm{Hz}}^{2}$; 0.5 ${\mathrm{Hz}}^{2}$]). The value of unknown detection probability $p_{D,k}\left(x\right)$ is first estimated by the corresponding pD-CPHD filter, and then bootstrapped into the GLMB filter. Clutter follows a Poisson RFS with average rates $c_{1}$ and $c_{2}$, as mentioned in Table 3. It can be seen that the tracker can precisely track the targets in latitude and longitude with respect to the time. There are some delays and missed detection in observations, which may be due to the distances from the estimated positions and the actual positions. The tracker shows the effectiveness of the tracking algorithm when the targets are merged or close together.

**Figure 6:**
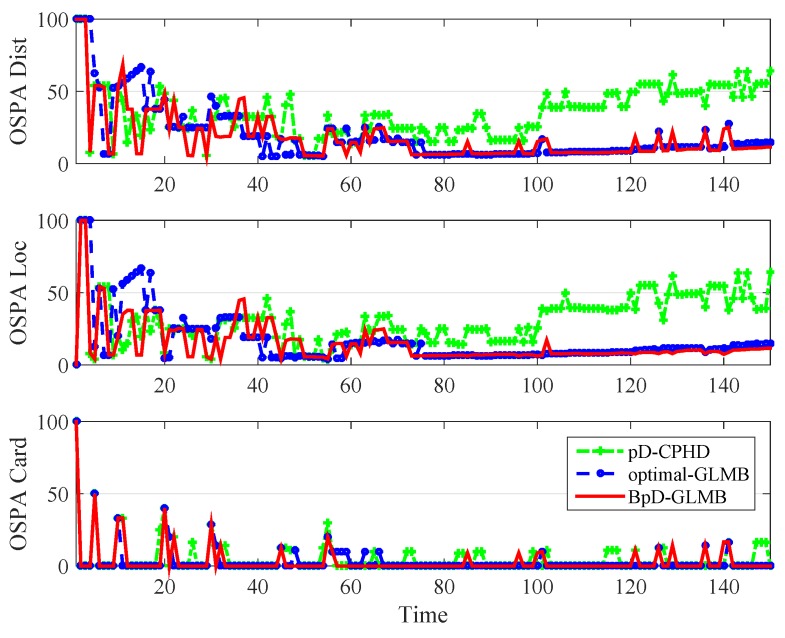
Comparision of Optimal Sub-Pattern Assignment (OSPA) among $p_{D}$ Cardinalized Probability Hypothesis Density (CPHD), GLMB, and bootstrapped $p_{D}$ GLMB.

**Figure 7:**
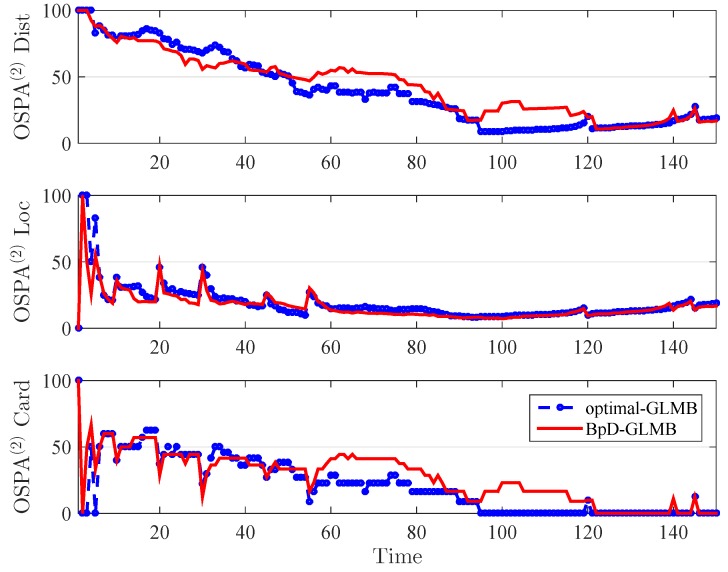
Comparision of OSPA ${}^{\left(2\right)}$ between GLMB and bootstrapped $p_{D}$ GLMB.

**Figure 8:**
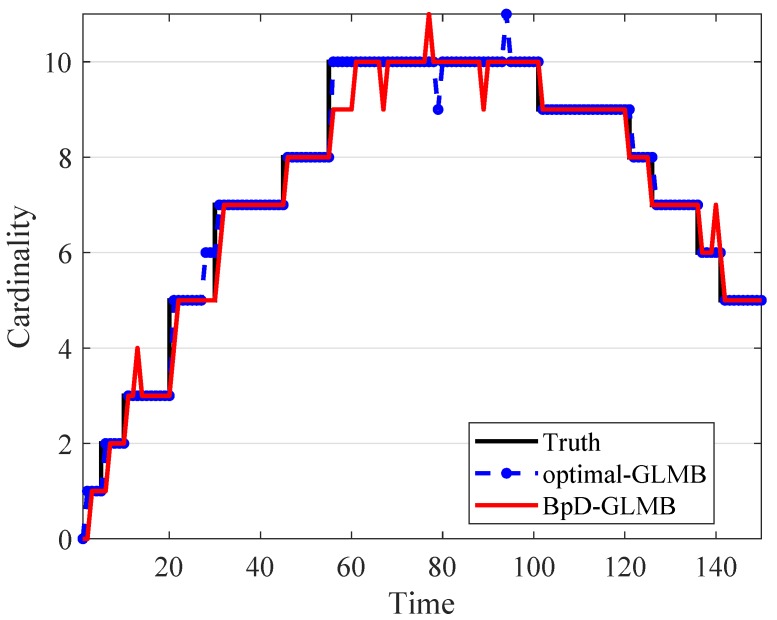
Comparison of target cardinality estimate between the GLMB and BpD-GLMB.

In this paper, the tracking errors of the filters are evaluated and compared using both the Optimal Sub-Pattern Assignment (OSPA) [57] and the OSPA− on −OSPA, or $OSPA^{\left(2\right)}$[36] with cut-off parameter $c_{0}=100$ and p=1. The OSPA metric is a distance between two sets of points that jointly account for the dissimilarity in the number of points and the values of the points in the respective sets. By using OSPA metric, the errors between the true and estimated multitarget states at each time step is calculated and shown in Figure 6. The OSPA− on −OSPA, or $OSPA^{\left(2\right)}$, distance has a different interpretation to that of the traditional OSPA distance. The $OSPA^{\left(2\right)}$ metric used in both GLMB and BpD-GLMB to capture the errors between the true and estimated sets of tracks over a period of time with window length set at l=20 is illustrated in Figure 7. In this paper, we assumed that the GLMB with known $p_{D}=0.98$ as the optimal filter. Further, a comparison of estimated cardinality between the GLMB and BpD-GLMB is shown in Figure 8. The results demonstrate the capability of our proposed BpD-GLMB filter, which outperformed the pD-CPHD filter, and its comparability to the GLMB filter with known $p_{D}$ in tracking accuracy under OSPA and $OSPA^{\left(2\right)}$ metrics.

## 5. Conclusions

An online multiple targets tracking filter with multistatic Doppler measurements was given in this work. The unknown probability of detection parameter, which was assumed to vary slowly compared to the data rate, was estimated and bootstrapped into the state-of-the-art GLMB filter to output target tracks. The simulation results demonstrate the effectiveness of the proposed method, the BpD-GLMB, which is comparable to the optimal-GLMB with a known detection probability while surpassing pD-CPHD significantly. Since the application only involves a small number of targets, the Murty’s algorithm was applied for the prediction and update performance of the tracker. For significantly faster implementation with a larger number of targets scenario, the joint prediction and update introduced in Reference [52] using Gibbs sampling could be used. Evidence has been shown in Reference [36] that the GLMB filter can be used to track over one million targets per scan in real time based on a $C^{++}$ platform. For strategies of realistic large-scale tracking implementation, MATLAB code is obviously insufficient, and it should be converted into $C^{++}$ or Python. The problem of tracking mobile targets using movable sensors will be further investigated for upcoming consideration.

## Figures and Tables

**Table 1:** Birth–death and dynamic model parameters.

Parameter	Symbol	Value
Sample period	Δ	0.15 (h)
Std. of the velocity noise	$\sigma_{v}$	0.3 (kn)
Std. of the course noise	$\sigma_{\phi }$	π/180 (rad $s^{-1}$)
Common existence probabilities	$\left({p_{B}}^{1,2};p_{B}^{3,4}\right)$	(0.01;0.02)
Survival probability	$P_{S}$	0.98
Number of targets	*N*	10

**Table 2:** Initial values of the targets.

Target	Init. Position	Init. Speed (kn)	Init. Course (rad/s)	Time of Birth (h)	Time of Death (h)
T1	$\left[17^{\circ }15^{\prime }15.66^{\prime \prime }N,110^{\circ }45^{\prime }06.84^{\prime \prime }E\right]$	[32, −5]	ψ/8	1	150
T2	$\left[14^{\circ }37^{\prime }52.46^{\prime \prime }N,113^{\circ }05^{\prime }43.44^{\prime \prime }E\right]$	[13, −9]	−ψ/2	5	120
T3	$\left[17^{\circ }09^{\prime }52.45^{\prime \prime }N,113^{\circ }05^{\prime }43.44^{\prime \prime }E\right]$	[−18, 5]	−ψ	10	140
T4	$\left[14^{\circ }37^{\prime }52.46^{\prime \prime }N,112^{\circ }53^{\prime }30.84^{\prime \prime }E\right]$	[2, 32]	−ψ/4	20	150
T5	$\left[18^{\circ }17^{\prime }11.54^{\prime \prime }N,107^{\circ }39^{\prime }48.60^{\prime \prime }E\right]$	[6, −20]	ψ/6	20	150
T6	$\left[18^{\circ }23^{\prime }47.54^{\prime \prime }N,107^{\circ }43^{\prime }24.60^{\prime \prime }E\right]$	[−12, −4]	ψ/4	30	140
T7	$\left[17^{\circ }10^{\prime }27.66^{\prime \prime }N,110^{\circ }42^{\prime }06.84^{\prime \prime }E\right]$	[15, −30]	ψ/8	30	130
T8	$\left[14^{\circ }38^{\prime }28.46^{\prime \prime }N,113^{\circ }05^{\prime }43.44^{\prime \prime }E\right]$	[−15, 30]	−ψ/2	45	135
T9	$\left[17^{\circ }14^{\prime }40.45^{\prime \prime }N,113^{\circ }05^{\prime }43.44^{\prime \prime }E\right]$	[28, −30]	−ψ/3	55	150
T10	$\left[14^{\circ }37^{\prime }52.46^{\prime \prime }N,112^{\circ }53^{\prime }30.84^{\prime \prime }E\right]$	[30, 5]	−ψ/4	55	150

**Table 3:** Measurement parameters.

Name	Symbol	Value
Transmitter	$p_{t}$	$\left[15^{\circ }22^{\prime }58.82^{\prime \prime }N,109^{\circ }07^{\prime }11.52^{\prime \prime }E\right]$
Receiver 1	$p_{r1}$	$\left[10^{\circ }22^{\prime }31.28^{\prime \prime }N,114^{\circ }28^{\prime }13.45^{\prime \prime }E\right]$
Receiver 2	$p_{r2}$	$\left[17^{\circ }58^{\prime }41.87^{\prime \prime }N,106^{\circ }24^{\prime }23.98^{\prime \prime }E\right]$
Transmit frequency	$f_{t}$	900 MHz
Speed of light	*c*	$3\times 10^{8}\left(m/s\right)$
Initial detection probabilities	[$p_{D1},p_{D2}$]	[0.70; 0.98]
Average clutter rate	[$c_{1};c_{2}$]	[10; 25]
